# A multifunctional conductive physiomimetic scaffold: synergy of rGO coating and cannabis-derived nanotopography for infection-resistant bone repair

**DOI:** 10.3389/fbioe.2026.1766388

**Published:** 2026-03-25

**Authors:** Fateme Hojaty Saeedi, Hosein Shahsavarani, Saadi Hosseini, Naser Farrokhi, Kajal Ghosal, Sabu Thomas, Atefeh Alipour, Pär K. Ingvarsson, Mehdi Jahanfar

**Affiliations:** 1 Department of Cell and Molecular Biology, Faculty of Life Sciences and Biotechnology, Shahid Beheshti University, Tehran, Iran; 2 Laboratory of Regenerative Medicine and Biomedical Innovations, Pasteur Institute of Iran, National Cell Bank, Tehran, Iran; 3 Nanofabrication and Tissue Engineering Research Laboratory, Department of Pharmaceutical Technology, Jadavpur University, Kolkata, India; 4 School of Chemical Sciences, Mahatma Gandhi University, Kottayam, Kerala, India; 5 Department of Nanobiotechnology, Pasteur Institute of Iran, Tehran, Iran; 6 Department of Plant Biology, Swedish University of Agricultural Sciences, Uppsala, Sweden; 7 Center for International Scientific Studies and Collaboration (CISSC), Ministry of Science, Research and Technology of Iran, Tehran, Iran

**Keywords:** antibacterial biomaterials, bone graft substitute, bone regeneration, cannabis trichomes, conductive scaffold

## Abstract

Conventional bone grafts cannot reliably fulfill the dual requirements of rapid osseoinduction and intrinsic infection-resistance to meet clinical needs. We therefore aimed to overcome this dual challenge by fabricating a novel physiomimetic three-dimensional scaffold. This was achieved by coating the unique nano-grooved cellulosic matrix derived from *Cannabis sativa* leaf trichomes with reduced graphene oxide (rGO) to mimic the native osteogenic niche. The plant-derived skeleton serves as a ready-made, topographically complex framework, while the rGO coating provides a microenvironment well suited for bone repair. Comprehensive characterization verified a measurable surface energy, hydrophilicity, roughness, and proper conductivity due to rGO coating. Moreover, *in vitro* examination confirmed that rGO biofunctionalization synergized with the innate nano-topography, dynamically accelerated the osteogenic differentiation of human adipose-derived stem cells. An upregulated expression of key bone markers, *COL1A1*, *RUNX2*, and *OPN*, sustained alkaline phosphatase activity, and augmented deposition of collagen and mineralized matrix exhibited the potential of the proposed approach for efficient osteal regeneration. An equally important finding was the scaffold’s inherent antibacterial property against Gram-positive and Gram-negative pathogens. We demonstrated that augmenting a natural cannabis-derived nanostructure with a conductive nanomaterial coating creates a multifaceted therapeutic strategy capable of promoting bone formation and potentially antibacterial effects, addressing two critical obstacles in regenerative orthopedics.

## Introduction

The emerging methodologies in so-called regenerative medicine via tissue engineering have begun to shed light on bone defects caused by trauma, tumors, or bone-related diseases (Nizan and Zulkifli, 2020). The field of tissue engineering is rapidly evolving, focusing on scaffold development and analyzing its potential for osteogenesis. The ideal scaffold should provide mechanical support to facilitate cellular attachment and proliferation, followed by differentiation into osteocytes with the potential to integrate into the surrounding bone tissue. Three-dimensional (3D) cultures, rather than two-dimensional (2D) cultures, provide a microenvironment more similar to the *in vivo* environment, thereby improving the *in vitro* development of organoids. Various platforms have been developed for the 3D culture of cells *in vitro* to aggregate and align cells, thereby resembling *in vivo* conditions, to enhance cell-cell communication and improve differentiation (Lee et al., 2019). Plant-based scaffolds, made from cell walls (mainly cellulosic polymers), have emerged as a promising alternative to conventional synthetic and animal-derived scaffolds due to their biocompatibility, bioavailability, sustainability, cost-effectiveness, lack of ethical concerns, minimal toxicity, and ease of modification (Shang et al., 2024; Galefi et al., 2023). These scaffolds have shown significant promise across various tissue engineering applications, with several studies highlighting their particular effectiveness in promoting bone regeneration (Lee et al., 2019).

The micro- and nanoscale architecture of these scaffolds can be engineered to mimic the native bone extracellular matrix (ECM), thereby promoting osteoblast adhesion and differentiation (Harris et al., 2021). Plant-based scaffolds can be surface-functionalized with osteoinductive agents to improve their mechanical properties, promoting the deposition of minerals critical for bone formation (Saberi et al., 2023; Salehi et al., 2020). The biodegradability of plant-based scaffolds offers a significant advantage in tissue engineering applications, as they degrade over time, allowing for gradual replacement of native bone tissue without the risk of chronic inflammation or the need for scaffold removal surgery. This characteristic renders plant-derived scaffolds highly attractive for osteogenesis, a process that depends on long-term scaffold integration for successful bone healing (Yun et al., 2023). Furthermore, the native structure of these extracellular matrices (ECMs) provides a foundation that can be combined with bioactive molecules, such as growth factors and proteins, to enhance the osteogenic process further (Li et al., 2024). For instance, in the study of Salehi et al. (2020), efficient osteogenesis from mesenchymal stem cells (MSCs) was confirmed by the overexpression of key osteogenic genes in decellularized spinach leaves (Salehi et al., 2020). Surface modifications of scaffolds with chemicals and small molecules have improved initial attempts at ECM development. For instance, decellularized watermelon rind coated with polydopamine enhanced surface adhesion, conductivity, and improved osteogenesis of MSCs (Banaeyan et al., 2023). In our other study, loading the date palm endocarp with proanthocyanidins caused enhanced osteogenic differentiation in adipose-derived mesenchymal stem cells via reducing oxidative stress (Galefi et al., 2023). Moreover, permeated proanthocyanidin on cellulosic 3D nanostructures increased alkaline phosphatase (ALP) activity and mineral deposition in dental pulp-derived mesenchymal stem cells (Hasanzadeh et al., 2024).

*Cannabis sativa* is famous for containing psychoactive and therapeutic metabolites and has been widely used for industrial hemp and medical purposes. Use of Cannabis as bioplastics (Merkle and Gerhards, 2024), nanofiber (Kaur et al., 2021) and textile material, bio-composites, and building material due to its lengthy, strong fibers (Jami et al., 2019), as commercialized under hempcrete (Malabadi et al., 2023), has proven its potential in other industries. It has a high capacity to absorb and retain water (Malabadi et al., 2023) in tissue engineering and its potential use in regenerative medicine. Cannabis fiber provides a rigid and durable structure due to its cellulosic content tightly interwoven with wall matrix polysaccharides, lignin, and pectic polymers (Dinçer Şahan et al., 2024). Cannabis trichomes are fine, hair-like structures on the cannabis plant that are responsible for producing and secreting economically important metabolites, mainly containing cannabinoids (Tanney et al., 2021). We used the Cannabis cell wall as a scaffold and compared it with a modified version incorporating reduced graphene oxide (rGO) to enhance the osteogenic differentiation of human mesenchymal stem cells (hMSCs). The morphology and physiochemical features of these two scaffolds were studied thoroughly. The osteogenic differentiation capacity of these scaffolds was evaluated by analyzing hMSC mineralization on scaffolds and osteodifferentiation-related gene expression.

## Materials and methods

### Materials

Sodium dodecyl sulfate (SDS), phosphate buffer saline (PBS: NaCl-KCl-Na_2_HPO_4_-KH_2_PO_4_), and glutaraldehyde were from Merck (Darmstadt, Germany). DMEM/Hams-F12, L-glutamine, and HEPES were obtained from Capricorn (Düsseldorf, Germany). cDNA and SYBRgreen kits were purchased from Pars Toos (Tehran, Iran). Fetal bovine serum (FBS) was bought from Gibco Thermo Fisher Scientific (New York, USA). Radioimmunoprecipitation assay (RIPA) buffer and Triton X100 were from DNA Biotech (Tehran, Iran). Chloroform and isopropyl alcohol were obtained from Sigma Aldrich (Oakville, Canada). 3-(4, 5-dimethylthiazolyl-2)-2, 5-diphenyltetrazolium bromide (MTT) powder and triazole were purchased from NTA (Tehran, Iran). 4′,6-diamidino-2-phenylindole (DAPI) color was from Life Technologies (Carlsbad, California, USA). Trypsin and antibiotics were from Biochrome (Berlin, Germany). Ethanol was purchased from Kimia alcohol Zanjan Co. (Zanjan, Iran).

### Cannabis leaf decellularization

Fresh Cannabis leaves covered with trichomes were collected and washed three times with distilled water (dH_2_O), then immersed in 10% (w/v) SDS for 7 days, with fresh SDS added every 2 days. Decellularized Cannabis scaffolds (DCS) were washed with dH_2_O for 3 h and immersed in 1% triton X100-sodium hypochlorite solution for 12 h. The DCS was gently washed 3 times with dH2O for 2 h each, then with PBS for 15 min. The DCSs were freeze-dried and stored at 4 °C.

### DNA isolation and quantification

DNA content of Cannabis was quantified before and after decellularization. Cannabis (100 mg) and DCS (100 mg) were weighed and pulverized in liquid nitrogen. Heated CTAB buffer (1 mL; at 60 °C) and 500 µL 1.0% β-mercaptoethanol were added to the mixtures and incubated for 30 min at 65 °C and then at 4 °C. Chloroform (500 µL) and isoamyl alcohol (20 µL) were added to the mixture and shaken vigorously for 2 min, followed by centrifugation at 12,000 rpm for 15 min. The supernatant was moved to a new microtube with the same amount of isopropyl alcohol for 1 h at −20 °C, and centrifuged at 12,000 for 15 min. The supernatants were removed, and pellets were dissolved in 700 µL of 70% ethanol and centrifuged at 12,000 rpm for 5 min. The supernatants were removed, and the pellets were air-dried and dissolved in 30 µL dH_2_O. DNA concentrations were quantified at 260 nm using an ELISA reader (Bio-Rad, USA).

### rGO synthesis and scaffold preparation

The rGO synthesis was performed via two reactions: graphite was reduced to form GO, and rGO was made by lowering the GO. Briefly, GO was prepared from graphite; nano graphite powder was dispersed in a mixture of H_2_SO_4_/H_3_PO_4_ and KMnO_4_ for 6 h. H_2_O_2_ was added to reduce manganese ions to soluble manganese sulfate and manganese oxides, and stirred for 10 min. The mixture was centrifuged, and the GO was washed using HCl to remove sulfate ions. GO was dried, and the powder was collected. GO powder was dissolved in dH_2_O, and 3 g of ascorbic acid as a reducing agent was added to the mixture. This mixture was stirred for 30 min at 60 °C. The cooled mixture was centrifuged for 40 min at 4000 rpm to remove the supernatant. Excess H_2_O_2_ was added to the black paste to oxidize the remaining ascorbic acid by stirring at 60 °C for 30 min. The black product was collected by centrifugation at 4000 rpm, washed with ethanol, washed 3 times with dH_2_O, and dried in a 120 °C oven for 24 h. For surface modification, the synthesized rGO was coated onto the scaffolds using 96% ethanol as a dispersion medium, followed by freeze-drying. Regarding the concern about residual surfactants, the samples were subjected to repeated washing steps during fabrication to minimize any residues. As the cell viability results already confirm good biocompatibility, we believe this washing protocol was sufficient; However, direct quantification via methylene blue or LAL testing could be considered in future studies for absolute confirmation.

### Morphological and physiochemical characterization

Field-emission scanning electron microscopy (FE-SEM) (TESCAN model MIRA3, Czech Republic and USA) and atomic force microscopy (AFM; Easyscan2 Nano surf, Netherlands) were used to identify surface morphology and topography. X-ray diffraction (XRD) and Fourier transform infrared spectroscopy (FTIR; BrukerTensor 27, Germany) were used for chemistry and crystal characterization.

Porosity, pore size, and specific surface area of the samples were measured using Brunauer–Emmett–Teller (BET) (BET Belsorp mini ll, Japan), and the porosity of the samples was calculated based on density. Square pieces of the samples were cut. Their density, thickness, and mass were determined by a vernier calliper and balance. The samples’ apparent density was quantified using volume and mass. These values were Equation 1 (Homaeigohar et al., 2021).

$$Ɛ=\frac{\left(\rho_{0}-\rho \right)}{\rho_{0}}\times 100$$ (1)

Ɛ is porosity% and $\rho_{0}$ is the bulk density of pure cellulose (1.5 g/cm^3^), and ρ is the bulk density of the sample.

The surface area is calculated by analyzing the amount of gas required to form a monolayer, using the Brunauer–Emmett–Teller (BET) equation. To determine pore volume and pore size distribution, the gas pressure is then increased incrementally until all pores are filled with liquid nitrogen. After that, the pressure is gradually reduced, allowing the condensed gas to evaporate from the system. Evaluating the resulting adsorption and desorption isotherms gives the necessary information regarding pore volume and size distribution. The specific surface area of the prepared scaffold was quantified using Brunauer–Emmett–Teller (BET) analysis. For this purpose, a BET plot was constructed within the standard relative pressure range of 0.05–0.35. Applying the BET equation enabled us to determine the monolayer-adsorbed gas volume (Vm) and the BET constant (C), which were then used to calculate the specific surface area (SBET) as defined in Equation 2.

$$S_{BET}=\frac{V_{m}\times N_{A}\times a_{cs}}{M\times m}$$ (2)

where N_A_ is Avogadro’s number, M is the nitrogen molecular mass, a_cs_ is the cross-sectional area of the adsorbate gas, and m is the mass of the sample. This widely adopted method is crucial for optimizing scaffold properties to ensure the necessary porosity and surface area for effective tissue regeneration (Brunauer et al., 1938; Bardestani et al., 2019). In addition to surface area, we evaluated the surface hydrophilicity by measuring the water contact angle after depositing a droplet onto the samples. To obtain a detailed topographical profile, Atomic Force Microscopy (AFM) was employed. This technique provides critical quantitative parameters, including the average roughness, root mean square (RMS) roughness, skewness, and kurtosis, which collectively describe the material’s surface characteristics. The extent of surface modification was quantified explicitly by calculating the average roughness (Ra) according to Equation 3.

$$R_{a}=\frac{1}{n}\sum_{i=1}^{n}\left|z_{i}-\underline{z}\right|$$ (3)

where $z_{i}$ represents individual height measurements and $\underline{z}$ is the mean height. Similarly, RMS roughness (R_q_) is calculated as Equation 4, providing a measure of the surface’s standard deviation.

$$R_{q}=\sqrt{\frac{1}{n}\sum_{i=1}^{N}{\left(z_{i}-\underline{z}\right)}^{2}}$$ (4)

Skewness ( $S_{k}$ ), which indicates the asymmetry of the surface height distribution, is calculated using Equation 5.

$$S_{k}=\frac{1}{N}\sum_{i=1}^{N}{\left(\frac{z_{i}-\underline{z}}{R_{q}}\right)}^{3}$$ (5)

Kurtosis (K), which describes the peakedness of the height distribution, is given by Equation 6 (Xie et al., 2024; Phupewkeaw et al., 2024).

$$K=\frac{1}{N}\sum_{i=1}^{N}{\left(\frac{z_{i}-\underline{z}}{R_{q}}\right)}^{4}$$ (6)

### Protein adsorption analysis

Protein adsorption analysis is crucial for understanding the interaction between biomaterials and biological systems, particularly how proteins adhere to material surfaces, which influences cell attachment, proliferation, and overall biocompatibility. To quantify protein adsorption of DCS and rGO-DCS, the scaffolds were treated with ethanol, washed with PBS, and incubated in MEM containing 10% FBS (v/v) for 24 h at 37 °C. The adsorbed protein on the scaffold was extracted using RIPA lysate buffer. Protein adsorbed on the scaffold surface was quantified by measuring the optical absorbance and interpolating the values from a BCA calibration curve.

### Evaluation of antibacterial potential of surface modified scaffold

The antibacterial activity of the two scaffold groups was evaluated using standard bacterial strains, including *Staphylococcus aureus* (Gram-positive; ATCC 25923) and *Escherichia coli* (Gram-negative; ATCC 25922). Briefly, the bacterial strains were cultured in nutrient broth containing 10 g/L beef extract, 10 g/L peptone, and 5 g/L NaCl, and incubated at 37 °C for 18 h on a rotary shaker. The resulting bacterial suspension was then diluted with nutrient broth and transferred to sterile phosphate-buffered saline (PBS) to achieve a final concentration of approximately 1.5–3 × 10^5^ colony-forming units (CFU) per ml. Next, 0.1 mL of this diluted bacterial solution was spread onto agar plates. Three pieces of each scaffold type (DCS and rGO-DCS) were then placed on the inoculated agar plates. The plates were incubated at 37 °C for 24 h to allow bacterial growth. After incubation, images were captured to measure the zone of inhibition around each scaffold, indicating their antibacterial activity.

### Scaffold preparation for cell culture

The decellularized leaf samples were cut into pieces to fit the cell culture plate and sterilized by UV for 30 min on each side, and washed with PBS containing penicillin/streptomycin three times. Samples were incubated in DMEM/F12 medium for 24 h at 37 °C.

### Cell viability and adhesion

Adipose-derived mesenchymal stem cells were purchased from Iran National Cell Bank, Pasteur Institute of Iran. Cell viability and adhesion are key indicators of the biocompatibility of the scaffold. Non-coated cannabis cell wall scaffold and coated scaffold were analyzed using FE-SEM imaging, MTT assay and DAPI (4′,6-diamidino-2-phenylindole) staining. For this purpose, 14 samples of each decellularized scaffold and rGO-coated scaffold were prepared as above. To quantify the cells’ metabolic activity on days 1, 3, 5, and 7, MTT (2,5-diphenyl-2H- tetrazolium bromide) assays were performed on Human Foreskin Fibroblast (HFF) cells. For this purpose, 5 × 10^3^ cells were seeded into each well of a 96-well plate. MTT reagent (5 mg/mL) was added to each well and incubated for 4 h, allowing the viable cells to convert MTT into insoluble formazan crystals. DMSO (1 mL) was added to each well to dissolve the formazan crystals. The media were transferred to a 96-well plate, and their absorbance was measured at 570 nm in triplicate. After day 7, the scaffold was washed with PBS to remove unattached cells and fixed in 4% paraformaldehyde. Samples were dehydrated by ethanol dilutions for SEM imaging.

### Swelling behavior and *in vitro* degradability

Swelling behavior tests are used to evaluate the water uptake capacity of the scaffolds. Since cells live in a water-based environment, the swelling behavior of the scaffold plays an important role in cell growth. The swelling percentage was calculated by submerging a specified weight of both coated and uncoated scaffolds in sterile PBS at 37 °C. The scaffolds were weighed after 1, 2, 4, 8, 16, 24 and, 48 h of incubation at 37 °C in PBS. The surfaces of the scaffolds were gently wiped with clean paper and then weighed. Water uptake capacity was calculated using Equation 7:

$$swelling\%=\frac{\left(w_{z}-w_{d}\right)}{w_{d}}\times 100$$ (7)

In this, w_z_ is the scaffold weight in the wet state, and w_d_ is at the dry state.

Understanding how a biomaterial degrades over time within a physiological-like environments is critical for its application in medical implants, drug delivery, and tissue engineering. To characterize this hydrolytic degradation, we incubated both the DCS and rGO-DCS scaffolds in PBS at 37 °C for a period of up to 100 days, simulating a physiological environment. On days 10, 20, 30, 40, 50, 60, 70, 80, 90, and 100, the scaffolds were removed, washed with dH_2_O, air-dried, and weighed. The weight loss was quantified by measuring the degradation rate of the scaffolds using Equation 8.

$$Degradation\;\%=\frac{\left(w_{o}-w_{l}\right)}{w_{l}}\times 100$$ (8)

W_l_ and w_o_ represent the primary and secondary weights of the scaffolds, respectively.

### Osteogenic differentiation analysis

A flask of the hMSC cell line was cultured, and the cells were enzymatically detached and suspended in DMEM containing 10% FBS and 1% penicillin/streptomycin. Cells (5 × 10^4^) were seeded on each sample and incubated at 37 °C under standard conditions. DMEM including 10% FBS and 1% penicillin/streptomycin was used as medium for standard samples, and this medium, plus 12 M dexamethasone, 50 g/mL ascorbic acid, 10 M β-glycerol phosphate, and bone morphologic proteins. Samples TCPS+, DCS+, and rGO-DCS+ received osteogenic supplement, DCS and rGO-DCS did not. Every 2 days, half the medium was replaced with fresh medium. At the end of 7, 14, 21, and 28 days after seeding, the cell-seeded scaffolds were washed with PBS, and some were then fixed in 4% paraformaldehyde at 4 °C for 24 h to preserve cell morphology. Samples were dehydrated using an ethanol series (20, 40, 60, 80, and 96%) for 10 min each. These samples were used for SEM imaging.

### ALP activity

Alkaline phosphatase (ALP) activity is a biochemical marker used to assess osteogenic differentiation of stem cells, as ALP is an early marker of bone formation. After washing samples with PBS, RIPA lysate buffer was added to some wells. The solutions, which now contained cells, were transferred to microtubes. These samples were centrifuged at 12,000 rpm for 15 min at 4 °C. The supernatants were transferred to a 96-well plate in triplicate. The ALP enzyme activity was measured at 405 nm using a microplate reader (BioTek Epoch, Santa Clara, CA, USA). ALP activity was normalized to the total protein content.

### Biomineralization

Bio-mineralization activities of hMSCs on DCSs and rGO-DCSs were measured by the Alizarin-red staining method (Bernar et al., 2023). At 7, 14, 21, and 28 days after cell culture, wells were washed with PBS, and cells were fixed in 4% paraformaldehyde. Samples were stained for 6 min with alizarin red (2%, w/v) at 22 °C. Samples were washed using dH_2_O, and the scaffold surfaces were imaged using an inverted phase contrast microscope (Olympus, CKX41, NY, USA). Calcium deposition was quantified by dissolving the dye in a 3% acetic acid aqueous solution and reading at 540 nm using a microplate reader.

### Quantitative real-time PCR

TRIzol reagent was used to isolate total RNA from cells at days 7, 14, 21, and 28 according to the protocols from German Cancer Research Center (DKFZ) and UConn Health. cDNA was synthesized by a cDNA synthesis kit according to the manufacturer’s protocol. Quantitative real-time PCR (qRT-PCR) was used to measure the relative expression of the key osteogenic genes. The expression levels of *COL1A1* (collagen type 1 alpha 1 chain), *BGLAP* (bone gamma-carboxyglutamate; human osteocalcin), and *SPP1* (secreted phosphoprotein 1) were measured using SYBR-green master mix and real-time PCR (RT-PCR, Carlsbad, USA) according to the manufacturer’s protocol. We normalized the expression levels of key osteogenic genes, including *COL1A1*, *SPP1*, *BGLAP*, and *RUNX2* using *GAPDH* (glyceraldehyde-3-phosphate dehydrogenase) as a reference. The primer sequences and corresponding conditions for this analysis are provided in Table 1. The selected genes are critical markers of bone formation: *COL1A1* encodes the principal collagen that provides the bone matrix with its structural integrity (Kannan et al., 2020). SPP1 (osteopontin) contributes to bone remodeling by facilitating osteoclast adhesion to the mineral matrix (Mazzoni et al., 2020). At the same time, *BGLAP* (osteocalcin) is a key osteoblast-secreted protein that supports bone mineralization through its affinity for calcium (Abduljauwad et al., 2023). *RUNX2* is a key transcription factor that regulates the expression of various osteogenic genes, orchestrating the differentiation of mesenchymal stem cells into osteoblasts and ensuring proper bone formation (Komori, 2022). The interplay among these genes is fundamental to the development of functional bone tissue for engineering applications. For real-time PCR (20 µL), Taq 2× Premix (10 µL), cDNA Control primer mix (1 µL), cDNA (1 µL), and 8 µL of PCR Grade Water were added to a RNase-free tube. PCR initiated with 1 cycle for 4 min at 95 °C and 35 cycles of [94 °C:30 s; 57 °C:30 s; 72 °C:30 s] and 1 cycle for 5 min at 72 °C.

**TABLE 1:** Sequences of forward (F) and reverse (R) primers, melting temperatures, and the product sizes for the corresponding genes.

Gene	Primer sequence (5'→3′)	Melting temperature (Tm ˚C)	Amplicon size (bp)
GAPDH	F: GTC TCC TCT GAC TTC AAC AGC G	64	129
	R: CAC CCT GTT GCT GTA GCC AA	60	
BGLAP	F: TCA CAC TCC TCG CCC TAT TG	60	133
	R: CTC TTC ACT ACC TCG CTG CC	63	
COL1A1	F: CAT CTC CCC TTC GTT TTT GAC	59	149
	R: CCA AAT CCG ATG TTT CTG CTG	59	
RUNX2	F: AGA TGA TGA CAC TGC CAC CTC	61	125
	R: GGG ATG AAA TGC TTG GGA ACT	59	
SPP1	F: GAG GTG ATG TCC TCG TCT GAT G	64	111
	R: CAC ATA TGA TGG CCG AGG TG	60	

### Immunostaining

Immunostaining with an anti-osteocalcin antibody (OC4-40) was used to detect the presence and the distribution of osteocalcin, a non-collagenous protein of bone matrix, as a marker of late-stage osteogenesis (Tsao et al., 2017). Immunostaining analysis was performed on the cell-seeded scaffolds after day 28 of culture. Samples were washed twice using PBS at 25 °C and fixed with 4% paraformaldehyde at 4 °C for 24 h. Samples were washed three times with PBS at 25 °C, 10 min each wash. They were washed for 5 min with 0.5× Triton X100 for the permeability analysis at 37 °C. The samples were washed with tap water and placed in PBS for 5 min. OC4-40 (1:150) was dropped into wells, and the samples were washed once with fresh PBS for 10 min. A secondary antibody (1:150), a goat anti-mouse FITC-conjugated,was added to the wells and incubated at 25 °C for 60 min. Samples were washed with running PBS and placed into fresh PBS for 10 min. DAPI was used to stain nuclei of the fixed cells for 30 min at 25 °C. The wells were washed 3 times with PBS for 10 min each at 25 °C. Samples were dried using ethanol serial dilution and analyzed using a fluorescence microscope.

### Statistical analysis

All experiments were performed in triplicate, and data are presented as mean ± standard deviation (SD). Statistical analysis was conducted using GraphPad Prism version 8. A sample size of n = 3 was used per group. Experimental results were normalized against the corresponding controls selected for each individual test. For gene expression analysis, target gene levels were normalized to GAPDH as the housekeeping gene. Statistical comparisons among groups were performed using one-way ANOVA followed by appropriate *post hoc* tests. A p-value <0.05 was considered statistically significant.

## Results and discussion

Currently, more than 100,000 patients are on the US organ transplant waiting list (Lewis and Cendales, 2021). The shortage of donor organs, associated immune rejection, and the use of immunosuppressive drugs continue to limit long-term patient transplant survival, leading to the need for tissue engineering (Crespo-Leiro et al., 2015). Autologous cell transplantation offers a promising solution to several critical challenges, including host rejection, the need for lifelong immunosuppressive drugs in patients, and the scarcity of available cell resources (Somasekhar and Griffiths, 2023). For tissue regeneration, biomaterials like tethered cellulose microfibrils have been widely explored as foundational scaffolds that ought to be biocompatible, biodegradable, and feature non-cytotoxic ECM-mimicking structures (Aghazadeh et al., 2022). Prior studies were mainly focused on synthetic electrospun cellulose together with either GO alone (Liu et al., 2017; Patel et al., 2019) or with some other compounds, such as hydroxyapatite (Ramani and Sastry, 2014). Modifications of synthetic scaffolds with GO resulted in better viability of the cells (Ramani and Sastry, 2014), improved biomineralization (Liu et al., 2017), enhanced cell proliferation and osteogenesis (Patel et al., 2019), and better physical properties such as wettability, roughness, yield, and higher cell proliferation rates (Kashte et al., 2020). Here, a decellularized Cannabis leaf cell wall was used to provide an ECM for osteogenic differentiation. Furthermore, rGO was used to modify the ECM surface due to its reported superior characteristics such as being cheaper than graphene, having high water solubility, zero bandgap, and excellent electronic, mechanical, and thermal properties (Ahmed et al., 2023).

Pro-inflammatory proteins, such as cytokines and chemokines, play a crucial role in the body’s immune response by promoting inflammation during infections or tissue damage. However, excessive production of these proteins can lead to chronic inflammation and various diseases. The modulation of pro-inflammatory proteins is a key focus of recent therapeutic research, with studies revealing insights across different biological contexts.

### GO anti-inflammatory effects

Bałaban et al. (2023) demonstrated that graphene oxide (GO) can lower pro-inflammatory protein levels in human skeletal muscle cells challenged with the SARS-CoV-2 spike protein, suggesting GO’s potential for anti-inflammatory effects to mitigate cytokine storms (Bałaban et al., 2023). This aligns with a broader understanding of inflammatory drivers.

### Key mechanisms

C-reactive protein (CRP), for instance, promotes inflammation by reprogramming glycolysis in human macrophages, thereby enhancing their pro-inflammatory activity (Newling et al., 2019). The inflammatory cascade is further amplified by mechanisms such as peroxynitrite-modified DAMPs, which enhance signaling through Toll-like receptor 4 (TLR4) and NF-κB (Zie et al., 2020). Central to this regulation are inflammasomes such as NLRP3; their activation promotes the secretion of potent cytokines, including IL-1β, that drive numerous inflammatory diseases (Strowig et al., 2012).

### IL-6 pathway

Furthermore, the interleukin-6 (IL-6) family modulates inflammatory responses in disease and cancer, particularly through IL-6 trans-signaling via the STAT3 pathway (Jones and Jenkins, 2018). The systemic impact of such inflammation is highlighted by findings that elevated plasma levels of inflammatory proteins correlate with the severity of Alzheimer’s disease, reinforcing the link between peripheral inflammation and neurodegeneration (Leung et al., 2013).

### ENGO effects on osteoclasts

In a study, nanographene oxide (ENGO) promoted angiogenesis and increased platelet-derived growth factor levels in preosteoclasts. *In vitro* experiments demonstrated that ENGO inhibited receptor activator of nuclear factor-kappaB ligand (RANKL)-induced osteoclast differentiation and bone resorption activity. Our results point to isocitrate dehydrogenase 1 (IDH1) as a central mediator.

### Molecular mechanism

We found that diminished IDH1 expression subsequently reduced the levels of histone lysine demethylase 7 A (KDM7A), which, in turn, resulted in an accumulation of H3K9me2 marks on the cathepsin K promoter region. These findings suggest that ENGO holds promise for applications in bone tissue engineering by modulating osteoclast–endothelial cell interactions, offering a potential strategy for treating bone resorption and osteoclast-related bone loss diseases (Liu et al., 2024).

### DNA content of the scaffold

**FIGURE 1:**
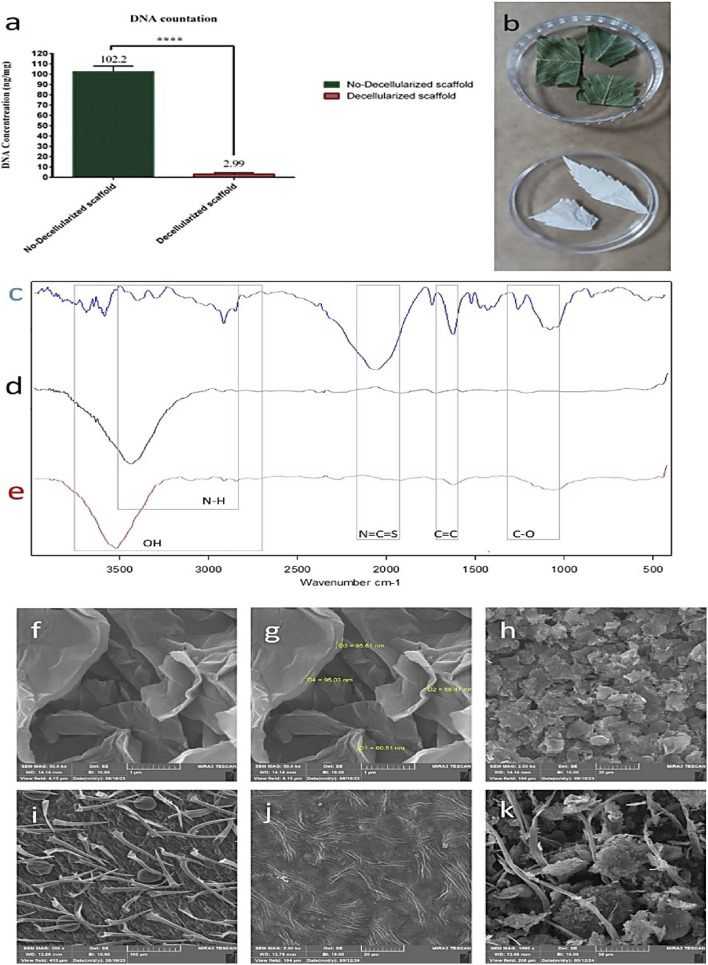
**(a)** DNA content of scaffold before (green) and after (red) decellularization. **(b)** Cannabis before and after decellularization. **(c)** ATR-FTIR spectra of DCS, **(d)** rgo, and **(e)** rGO-DCS indicate the increase of -OH groups due to rGO coating. Peaks at 2700–3,700 cm^−1^ represent hydroxyl groups and also N-H bonds, a peak at 2050 cm^−1^ represents N=C=S bonds, a peak at 1616 cm^−1^ (peak range of 1610–1678 cm^−1^) indicates C=C bonds, and two peaks at 1065–1245 cm^−1^ indicate the C-O bonds. The broad peak at 3,448 cm^−1^ indicates O-H bonds of rGO. The broad peak at 3,529 cm^−1^ indicates O-H bonds, and two peaks at 1062 cm^−1^ indicate C-O bonds. **(f–h)** the Fe-SEM images of rGO show nanosheets of rGO **(i)** Cannabis before decellularization **(j)** Cannabis after decellularization shows the cellulosic fibers of Cannabis. The surface structure of Cannabis is revealed after decellularization. **(k)** rGO-DCS scaffold image shows the presence of rGO nano sheets on the scaffold.

Decellularization was checked by controlling DNA content in the prepared ECMs (Figure 1a). At the beginning, the average DNA content of Cannabis was 102.2 ng/mL, which significantly declined to 2.99 ng/mg after decellularization.

### Chemical structure and surface morphology analysis

The surface chemistry of the DCS and rGO-DCS was characterized using FTIR spectroscopy (Figures 1c–e). The peaks at 2700–3,700 cm^−1^ represent hydroxyl groups and N-H bonds, the peak at 2050 cm^−1^ represents N=C=S bonds. The peak at 1616 cm^−1^ indicates C=C bonds, and two peaks at 1065–1245 cm^−1^ indicate C-O bonds. The broad peak at 3,448 cm^−1^ indicates O-H bonds of rGO. The broad peak at 3,529 cm^−1^ is attributed to O-H bonds. Two peaks at 1610–1678 cm^−1^ indicate the C=C bonds, and a peak at 1062 cm^−1^ indicates C-O bonds. FTIR (Emiru and Ayele, 2017) confirmed successful DCS coating with rGO. The surface morphology of the Cannabis scaffold before and after decellularization, and the rGO-DCS is shown in Figures 1i,j. The curved sheets of rGO on DCS are shown in Figures 1f–h. The Cannabis morphology is fibrillar, and fibers are aligned, forming half-circular shapes, after decellularization. The rGO-modified DCS surface exhibits increased roughness (Figure 1k).

### Surface topography

Specific surface area and porosity of the scaffolds were analyzed to describe liquid absorption behavior. BET analysis showed a surface area increase from 0.1209 (DCS) to 2.2767 (rGO-DCS) m^2^/g, while the mean pore diameter decreased from 70.7550 to 26.9020 μm. The total pore volume increased from 0.0021 to 0.0153 cm^3^/g. This indicates that the rGO coating has increased both the surface area and total pore volume.

### Roughness and cell adhesion

**FIGURE 2:**
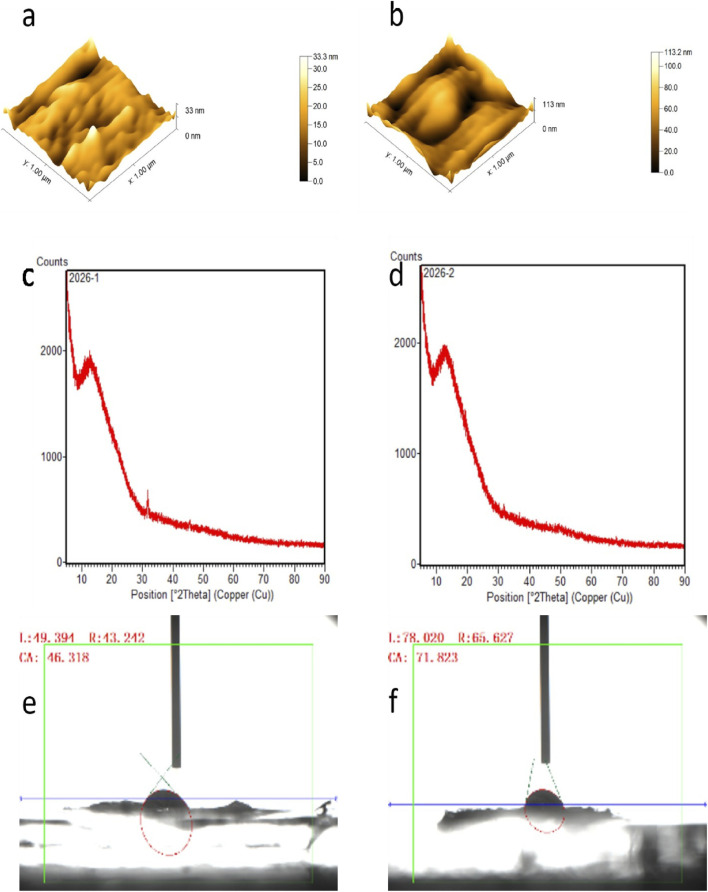
AFM analysis of **(a)** DCS and **(b)** rGO-DCS scaffolds. rGO-DCS has more roughness on the surface compared to rGO-DCS. XRD of **(c)** DCS and **(d)** rGO-DCS shows the peaks related to cellulose and rGO. Contact angle measurements of **(e)** DCS (46.31°) and **(f)** rGO-DCS (71.82°) indicate that both scaffolds are hydrophilic. Hydrophilicity decreased after coating with rGO.

The surface roughness, measured as the difference between the highest and the lowest point of DCS was 33.3 nm and for rGO-DCS was 113.2 nm, indicating increased roughness due to rGO coating (Figures 2a,b). This can facilitate better cell placement on the scaffold’s surface. rGO-DCSs pore diameter and specific surface area were lower than date endocarp coated with grape seed proanthocyanidin (Galefi et al., 2023), polydopamine watermelon rind (Banaeyan et al., 2023), and even solely spinach (Salehi et al., 2020). This may be because DCS is not inherently highly porous, unlike the others.

### Crystallinity and hydrophilicity analysis

We employed X-ray diffraction (XRD) to analyze the crystallographic structure of the scaffolds, a technique pivotal in bone tissue engineering for characterizing the phase composition and atomic arrangement of biomaterials (Özarslan et al., 2024). This analysis reveals how scaffold materials interact with their biological surroundings, informing the design of more effective bone regeneration strategies (Adry et al., 2024). In this context, the XRD diffraction patterns obtained for the rGO-DCS scaffold were nearly identical to those of the unmodified DCS control (Figures 2c,d). This indicates that their crystallinity is the same or very near to similar chemical composition. The XRD peaks of rGO and cellulosic structures were identical to those in earlier studies (Cao and Zhang, 2015). The hydrophilicity of DCS was greater than rGO-DCS (Figures 2e,f) as the contact angle was inclined by rGO surface modification from 46.31–71.82°. The contact angle results indicate that the hydrophilicity of DCS is comparable to watermelon rind, and rGO-DCS is almost the same as date endocarp (Galefi et al., 2023), and spinach (Salehi et al., 2020) all being hydrophilic.

### Cell viability and adhesion

**FIGURE 3:**
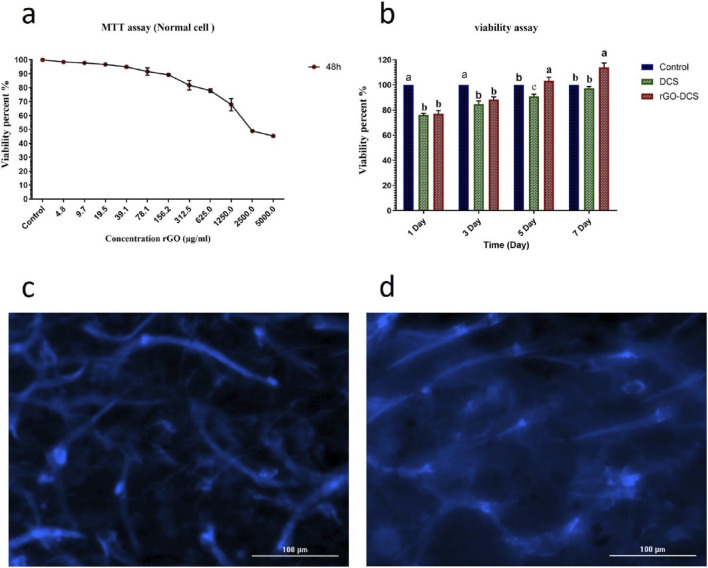
**(a)** Viability percentage in different concentrations of rGO. **(b)** viability assay on day 7. rGO-DCS demonstrated superior biocompatibility and cell growth. **(c,d)** DAPI staining images of DCS **(c)** and rGO-DCS **(d)**. In both scaffolds, live HFF cells are visible (light blue round shapes are cell nuclei).

HFF cell line was used to check the cell viability and adhesion of the scaffolds. DAPI staining showed proper cell attachment on both scaffolds (Figures 3c,d). The viability of the cells on each scaffold was analyzed using an MTT assay (Figure 3b). An initial viability decline was noted on day 1 (80%), due to the acclimatization of cells to the environment. On day 7, the viability increased to 98% for DCS and 118% for rGO-DCS, demonstrating the suitability of the ECMs. Nevertheless, the rGO-coated sample showed better performance. Additionally, MTT assay was used to determine the suitable concentration of rGO in DCS coating after 48 h (Figure 3a). As the concentration increased from 4.8 to 5000.0 μg/mL, cell viability decreased sharply, with a sharp decline at 39.1 μg/mL. Thus, an rGO concentration of 0.02 g/mL was used for DCS coating. The MTT results for DCS and rGO-DCS showed a higher percentage of living cells than date endocarp-grape seed proanthocyanidin (Galefi et al., 2023), spinach (Salehi et al., 2020), polydopamine-watermelon rind (Banaeyan et al., 2023), and synthetic cellulose scaffolds (Liu et al., 2017; Patel et al., 2019). Interestingly, bacterial cellulose yielded results comparable to those of DCS and rGO-DCS (Ramani and Sastry, 2014).

Prolonged decellularization with 10% SDS and Triton X 100 could leave trace amounts of detergents, therefore, directly quantify residual SDS/Triton (e.g., by methylene blue assay) or perform endotoxin testing (e.g., LAL). It is important to emphasize that future work will incorporate quantitative assays for residual detergents and endotoxin to exclude their confounding effects on cell viability and any putative immunomodulatory responses.

### *In vitro* swelling behavior and degradation

**FIGURE 4:**
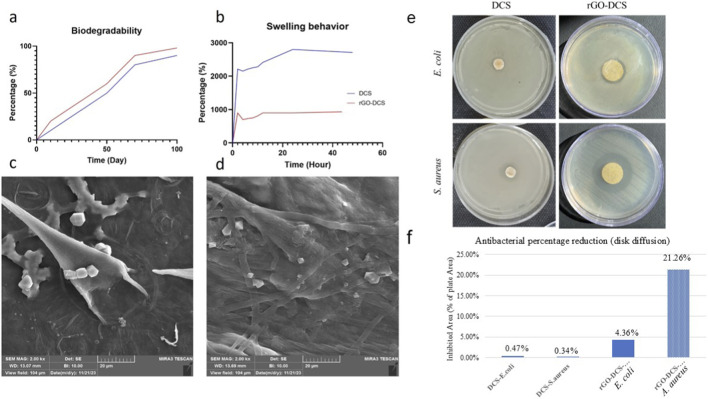
**(a)** The biodegradability of DCS and rGO-DCS indicates a good biodegradability of about 90% in 100 days. **(b)** The swelling behavior of DCS (blue line) was 2800% of its weight, and rGO-DCS is about 1000% of its weight. Fe-SEM imaging of **(c)** DCS and **(d)** rGO-DCS shows the scaffold degradation **(e)** Antibacterial assay DCS and rGO-DCS on gram-negative (*E coli*) and gram-positive (*S. aureus*) bacterial strains. The aura around each disc shows the inhibition of bacterial growth. **(f)** Data from the antibacterial assay in **(e)**.

Scaffolds are meant to degrade inside the body over time, leaving the differentiated cell type. For this purpose, *in vitro* degradation of scaffolds was analyzed for 100 days, showing 90% scaffold loss as illustrated by FE-SEM (Figures 4c,d), with higher degradation for rGO-DCS compared to DCS (Figure 4a). DCS exhibited a higher swelling ratio and water uptake capacity, possibly due to the resulting surface modification. After 50 h of scaffold immersion in PBS, DCS absorbed 28, and rGO-DCS absorbed about 10 times its weight (Figure 4b). rGO-DCS and DCS exhibited faster degradation rates and greater water absorption than in our earlier studies (Galefi et al., 2023; Salehi et al., 2020; Banaeyan et al., 2023).

### Antibacterial activity

Bacterial colonization and subsequent biofilm formation are major challenges in bone repair and orthopedic applications. For this reason, assessing the antibacterial activity of tissue-engineered constructs is an important aspect in bone regeneration systems (Thrivikraman et al., 2018). This phenomenon is related to bacterial infiltration along the implanted scaffold, which ultimately delays bone and tissue regeneration (McCaig et al., 2005). In this study, the antibacterial properties of the DCS and rGO-DCS scaffolds were assessed using the disk diffusion method against Gram-negative and Gram-positive bacterial strains. As shown in Figure 4, the established DCS and rGO-DCS scaffolds (Figures 4e–f) exhibited visible zones of inhibition against *S. aureus* and *E. coli*. However, as expected, the DCS scaffold produced a smaller inhibition zone than the rGO-DCS scaffold, as shown for both Gram-positive and Gram-negative bacterial strains, indicating the significant contribution of rGO incorporation to the antibacterial performance of this synthesized scaffold. Furthermore, for each scaffold, the antibacterial effect was greater against the *S. aureus* Gram-positive strain (Figure 4f) than the *E. coli* Gram-negative strain (Figure 4e) in both scaffold groups.

### Osteogenic differentiation

**FIGURE 5:**
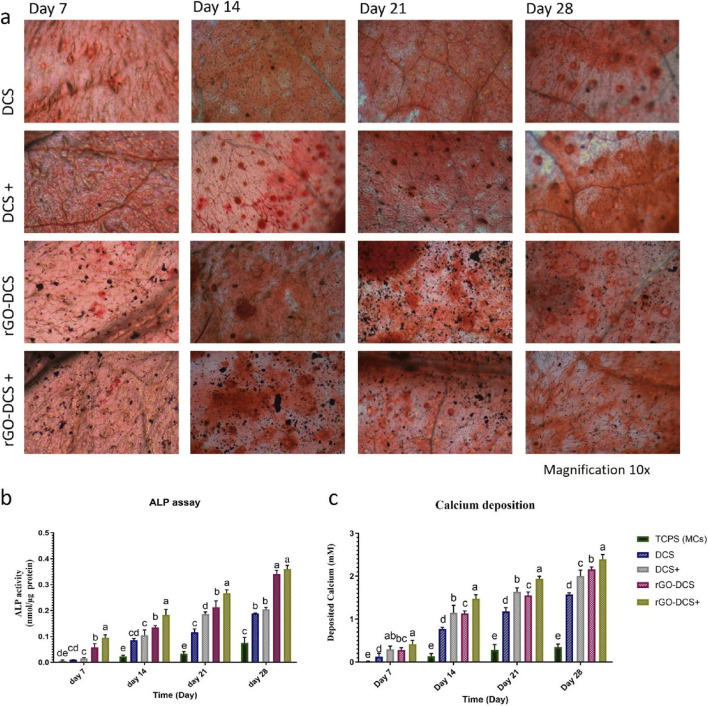
**(a)** Alizarin red staining shows an increase in calcium from day 7 to day 28 (magnification ×10), with a greater increase in rGO-DCS. **(b)** alkaline phosphatase activity increased from day 7 to day 28 of differentiation. ALP activity was higher in rGO-DCS compared to DCS and the control cells. **(c)** Calcium deposition had a higher rate in rGO-DCS in comparison to DCS and control cells. In both ALP activity and calcium deposition, positive controls, including DCS+ and rGO-DCS+, showed slightly higher rates than DCS and rGO-DCS.

To get a clearer picture of how well these materials support bone formation, we turned to Alizarin-Red staining. This method is quite useful for spotting calcium deposits, which are a solid indicator that cells are transforming into bone-like tissue. It allowed us to look at the mineralization process on the different scaffold groups, including the uncoated ones, the DCS, and the rGO-DCS versions, from both a visual and numerical standpoint. By running a calcium content assay at several points throughout the experiment, specifically on days 7, 14, 21, and 28, we could measure exactly how much mineral was being laid down (Figure 5a). Between the first and third weeks, both the DCS and rGO-DCS scaffolds showed a clear, steady increase in mineralization, indicating active bone cell activity. Where things got a bit more interesting was with the rGO-DCS scaffold. It consistently showed slightly higher calcium levels compared to the standard DCS, a difference that was especially visible at the 2-week and 4-week marks. This difference, while modest, hints that the graphene oxide coating might give the scaffold an extra nudge, possibly helping cells push further along in the bone-forming process during the later stages of maturation.

Moreover, DCS+ and rGO-DCS+ showed a significantly improved (*p* < 0.05) calcium deposition behavior compared to the DCS and rGO-DCS. , Observation of significant greater calcium deposition at all 7th, 14th, 21st, and 28th days of incubation in rGO-DCS+ and DCS+ suggest a synergistic consequence between the scaffolds and osteogenic media (Figure 5b). In contrast, the control without scaffolds, cultured on the plate, had the lowest calcium deposition rate.

### ALP activity

**FIGURE 6:**
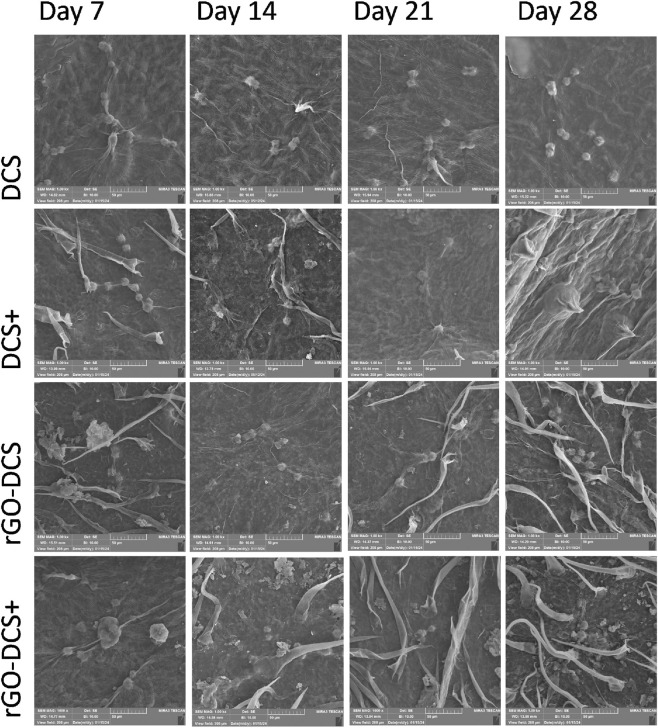
SEM imaging of cells during osteogenesis on DCS and rGO-DCS samples. Cell masses increased over time, and the cell population was higher beneath the trichomes, indicating that the trichomes physically support the cells.

ALP activity analysis (Figures 5c,b) indicated that the ALP activity for DCS and DCS+ has increased from 0.0095 to 0.0150 nmol/μg on day 7 to 0.1885 and 0.2050 nmol/μg on day 28, respectively. For rGO-DCS and rGO-DCS+, they were increased from 0.0578 to 0.0950 nmol/μg on day 7 to 0.3405 and 0.3600 nmol/μg on day 28. Additionally, changes in cell morphology and indication of osteogenic differentiation with larger cell sizes were observed in SEM images taken on days 7th, 14th, 21st, and 28th of incubation (Figure 6). ALP activity of rGO-DCS was higher than that of DCS. Furthermore, these established scaffolds exhibited higher ALP activity than in earlier reports (Galefi et al., 2023; Salehi et al., 2020; Banaeyan et al., 2023; Patel et al., 2019; Ramani and Sastry, 2014; Kashte et al., 2020). Since Alkaline phosphatase is highly expressed in bone, liver, and kidney tissues, its higher expression suggests differentiation into these tissues. To further corroborate osteogenesis, we performed other studies as follows.

### Immunostaining and fluorescence microscopy

**FIGURE 7:**
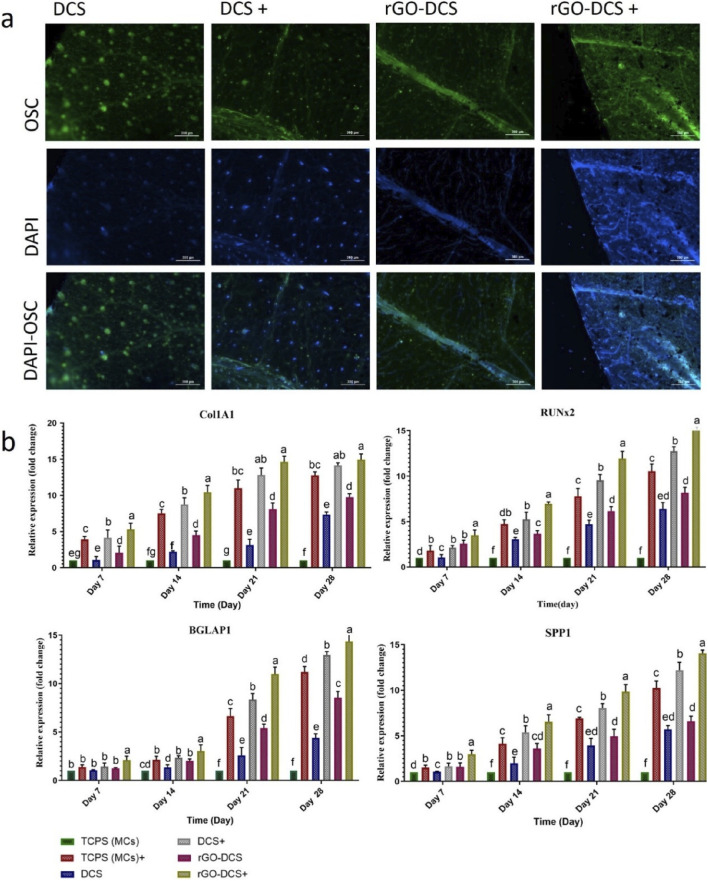
**(a)** Osteocalcin (OSC) immunostaining and DAPI staining of hMSCs on day 21 of osteogenic cell differentiation. The immunofluorescence staining images verify that the DCS and rGO-DCS can promote the expression of the *OSC gene drives* bone extracellular matrix development and matrix mineralization. **(b)** expression of *SPP1*, *Col1A1*, *BGLAP* and *RUNx2*. Expression of these genes increased significantly over the 28 days of osteogenesis. The scaffolds demonstrated the capability for cell attachment, growth, and osteodifferentiation. The highest increase belonged to rGO-DCS+. This indicates the efficiency of the rGO-DCS was higher than the others. Nourishing the cells with a medium containing osteo-inducer factors had a significant impact.

To monitor osteogenic activity and track cell proliferation, viability, and distribution within the engineered tissue, the osteocalcin immunostaining was used in conjunction with DAPI staining. Together, these techniques provide a comprehensive assessment of both the molecular and cellular dynamics of bone formation, offering critical insights that help advance the development of robust bone tissue engineering strategies (Sollazzo et al., 2010; Zhou et al., 2021). DAPI staining (blue) and osteocalcin immunostaining (green) on samples are shown in Figure 7a. The green dots indicate OSC detection in cells, whereas the blue dots represent live cells on both DCS and rGO-DCS scaffolds. Higher numbers of live cells and OSC were evident in positive samples. Overall, rGO-DCS showed higher OSC expression and more live cells. Scaffolds prepared from Cannabis had better performance than (Galefi et al., 2023).

### Expression of bone-specific genes

The expressions of *COL1A1*, *SPP1*, *BGLAP*, and *RUNX2* genes for different scaffolds are presented (Figure 7b). The gene expressions were measured on TCPS (tissue culture polystyrene, hMSCs), TCPS+, DCS, DCS+, rGO-DCS, and rGO-DCS+. Similarly, transcript analysis was performed at 7, 14, 21, and 28 days post-cell culture.

The expression level of *COL1A1* increased almost evenly from day 7–21 in all samples and stopped at that level from day 21 to day 28. Expression of this gene increased by about 10 units in each sample from day 7 to day 28 compared with TCPS. The rGO-DCS samples exhibited higher expression than TCPS and DCS. The comparison between DCS+ and rGO-DCS+ and DCS and rGO-DCS indicates that DCS and rGO-DCS showed a very high performance to their full potential. They had higher expression than TCPS+ and TCPS.

The expression level of *RUNX2* increased evenly from day 7 to day 28 and was primarily expressed in rGO-DCS+ followed by DCS+. Accordingly, the gene expression levels in rGO-DCS and DCS were comparable to those in TCPS+. They were all higher than TCPS on all days. The expression level of *SPP1* was comparable to that of *RUNX2*, with a consistent increase from day 7 to day 28. For *SPP1*, the expression level in DCS and rGO-DCS was almost the same. rGO-DCS showed slightly higher expression, compared to DCS and rGO-DCS. Similarly, the expression was slightly higher in rGO-DCS+ than in DCS+. These samples showed higher SPP1 expression in TCPS, rGO-DCS+, and DCS+ than in TCPS+.

The expression level of *BGLAP1* was almost steady for all samples from day 7 to day 14. Its expression increased significantly from day 14–21, with a slower trend from day 21–28. The expression of this gene was as rGO-DCS+ > DCS+ > TCPS+ > TCPS.

Overall, these genes showed higher expression in the presence of rGO, indicating a greater osteogenic differentiation in the presence of this construct. With a general pattern of expression as TCPS < DCS < rGO-DCS < TCPS+ < DCS+ < rGO-DCS+. In comparison with other studies, expression results showed that the expression level of *COL1A1* in polydopamine-watermelon rind and rGO-DCS was almost the same on day 21 and was higher than in spinach on day 18. The expression levels of *BGLAP1*, *RUNX2,* and *SPP1* were about the same in rGO-DCS, date endocarp-grape seed proanthocyanidin (Galefi et al., 2023), and polydopamine-watermelon rind (Banaeyan et al., 2023), although rGO-DCS was slightly better. Their expressions were higher than DCS. The expression level of *RUNX2* was higher than in spinach (Salehi et al., 2020). The upregulation of RUNX2, SPP1, and BGLAP expression in our conductive nano-grooved-scaffolds 3D cell culture, confirms the activation of osteogenic differentiation. Several studies have demonstrated that conductive biomaterials promote osteogenic gene expression by simulation focal adhesion dynamics and cytoskeletal tension through mechanotransductive pathways, such as involving FAK-mediated signaling (Wang et al., 2016; Sun et al., 2017). Furthermore, the biomaterial conductivity influences endogenous electrical signaling and enhance osteogenic differentiation even in the absence of an external electrical stimulation (Balint et al., 2014; Thrivikraman et al., 2018). Although, these signaling pathways were not directly analyzed in the this study, the subsequent investigation of RUNX2 and its downstream targets represent that similar mechano-electrical regulatory mechanisms which may contribute to the enhanced osteogenic response obtained in our system.

The underlying nano-grooved structure of Cannabis leaf trichomes can provide contact improvement-guidance. This specific topography offers contact guidance, which helps align integrin proteins, strengthens initial cell adhesion, and encourages anisotropic cell spreading. When cells stretch out in a directed manner, it leads to the formation of organized actin stress fibers and builds higher cytoskeletal tension, both of which are critical early signals for driving stem cells toward a bone lineage (Kim et al., 2009).

Conductive coating nanostructure, such as coating with rGO, introduces a conductive microenvironment at the basic scaffold surface, which can influence cellular behavior without externally applied electrical stimulation (Savchenko et al., 2021). A combination of integrated nano-grooved-structure scaffolds with the electrical properties of the conductive coating, creates specialized osteogenesis inducer niches that encourages osteogenesis which amplifying focal adhesion signaling and cytoskeletal-mediated lineage commitment.

In other words, this synergistic effect can drive localized charge redistribution via cell adhesion, cytoskeletal contraction, and ionic exchange (Thrivikraman et al., 2018). Moreover, mechanotransductive forces generated by cell attachment and detachment can generate endogenous electrical potentials that are redistributed across the conductive rGO layer (McCaig et al., 2005; Shin et al., 2016). The improved intercellular communication and more coordinated osteogenic responses can be partly attributed to the increased conductivity of the rGO-coated three-dimensional scaffold (Zhou et al., 2016).

Scaffold conductivity can also contribute to mineral deposition by affecting localized charge accumulation, ionic mobility, and the nucleation and growth of calcium phosphate minerals at the scaffold surface (Dubey et al., 2015) The strong osteogenesis properties, together with intrinsic antibacterial activity, introduce these nano-compatible bone biomaterials for cell alignment and orthopedic applications. This is particularly an urgent requirement in emergency clinical situations, such as the prevention of high-risk contaminated bone defects, open fractures, and infection in post-debridement reconstruction in osteomyelitis. Traditionally, these problems are managed in a two-step process, which includes controlling infection and reconstructing bone structure. This mechanism can prolong recovery and increase the risk of complications (Feng et al., 2023). Therefore, offering a dual-function scaffold provides an integrated approach that addresses infection control and bone regeneration simultaneously. Antibacterial activity can reduce early bacterial adhesion and biofilm formation, as well as support host cell survival and allow osteogenic cells to attach, spread, and interact with the scaffold during the early healing phase. Concurrently, osteogenesis arises from a series of interactions, including cell proliferation, differentiation, and extracellular matrix expansion, thereby enabling sustained bone regeneration even under infection-prone conditions. From a translational perspective, utilization of multifunctional platforms such as rGO-coated structures can control infection and regenerate bone, which may reduce dependence on extensive antibiotic therapy, decrease the need for secondary interactions, and recover structural and functional outcomes in high-risk orthopedic conditions (Cai et al., 2025).

## Conclusion

We addressed a persistent challenge in orthopedics by developing a scaffold that simultaneously supports rapid bone formation and resists bacterial colonization. Proposed approach combined the innate nano-grooved topography of a cannabis-derived cellulosic matrix with an improved conductivity via reduced graphene oxide coating. The plant-based skeleton provided a ready-made, complex architecture that mimics aspects of the native extracellular environment, while the rGO layer introduced a conductive, hydrophilic surface with measurable roughness and favorable surface energy. These physical and chemical properties together created a physiomimetic three-dimensional niche suited for bone repair. The combination of rGO biofunctionalization and the underlying nanotopography accelerated differentiation beyond what either feature might achieve alone. This was evident from the upregulated expression of key bone-related genes, including COL1A1, RUNX2, and OPN, along with sustained alkaline phosphatase activity throughout the culture period. Collagen deposition and mineralized matrix accumulation further confirmed that the scaffold supports the full sequence of osteogenic maturation, from early commitment to late-stage mineralization.

Having dual functionality in antibacterial effects against both Gram-positive and Gram-negative pathogens in addition to its bone-forming capacity, is particularly fit it for clinical scenarios such as open fractures or contaminated defects where infection risk complicates bone healing. By combining osteoinductive capability with infection-resistance in a bio-inspired single 3D nanoconstruct, this platform reduces the need for separate interventions and simultaneously can address current main challenges in regenerative orthopedics. In conclusion, the rGO-functionalized cannabis-derived scaffold offers a multifaceted therapeutic route toward bone repair, while bioinspired microenvironment is not only structurally supportive but also biologically instructive and inherently protective against microbial threats.

## Data Availability

The raw data supporting the conclusions of this article will be made available by the authors, without undue reservation.
